# Investigating brain maturation, neurodevelopmental and psychiatric outcomes in individuals with early-onset liver disease: protocol of a single-centre observational study

**DOI:** 10.1136/bmjopen-2025-103290

**Published:** 2025-11-26

**Authors:** Melanie Ehrler, Megan Earl, Jemma Day, Jonathan O’Muircheartaigh, Luke Mason, Nicolaas Puts, Flavio Dell’Acqua, Charlotte E Blackmore, Grainne M McAlonan, Marianne Samyn

**Affiliations:** 1 Department of Forensic and Neurodevelopmental Science, King’s College London Institute of Psychiatry Psychology & Neuroscience, London, UK; 2 Child Development Center, University Children’s Hospital Zürich, Zürich, Switzerland; 3 Maudsley Biomedical Research Centre, South London and Maudsley NHS Foundation Trust, London, UK; 4 Paediatric Liver, GI and Nutrition Centre, King’s College Hospital, London, UK; 5 Centre for the Developing Brain, School of Biomedical Engineering & Imaging Sciences, King’s College London, London, UK; 6 MRC Centre for Neurodevelopmental Disorders, King’s College London, London, UK; 7 Department of Neuroimaging, King’s College London Institute of Psychiatry Psychology & Neuroscience, London, UK; 8 National Adult ADHD and Autism Service, South London and Maudsley NHS Foundation Trust, London, UK; 9 Forensic and Neurodevelopmental Sciences, King’s College London Institute of Psychiatry Psychology & Neuroscience, London, UK

**Keywords:** Paediatric gastroenterology, Hepatobiliary disease, Developmental neurology & neurodisability, MENTAL HEALTH

## Abstract

**Abstract:**

**Introduction:**

Early-onset chronic liver disease (CLD) and its subsequent clinical progression have systemic impact. Its trajectory coincides with critical periods of brain development. In this study, we will test the hypothesis that early-onset CLD is associated with neurodevelopmental and psychiatric symptoms and delineate their neurobiological underpinnings through multimodal neuroimaging.

**Methods and analysis:**

This study will recruit 100 patients with biliary atresia and 50 patients with other types of early-onset CLD, aged between 6 and 30 years, under the primary care of Paediatric Liver Services at King’s College Hospital, London, UK. Cognitive performance and autism-related behaviours will be evaluated with neurodevelopmental assessments. Participants and their parents will complete questionnaires addressing neurodevelopmental and psychiatric outcomes in everyday life, and quality of life. Multimodal neuroimaging will be conducted using electroencephalography (EEG); eye-tracking; structural, functional and diffusion MRI; and magnetic resonance spectroscopy (MRS). Clinical information will be collected from patients’ medical records and bio samples. Data of 222 neurotypical controls and 307 neurodivergent controls without CLD will be pooled from the Longitudinal European Autism Project with a similar study protocol. Neurodevelopmental and psychiatric outcomes will be compared with normative values and between groups. Associations with clinical risk factors will be explored using multivariable regression. Neuroimaging markers will be compared between groups and associations with neurodevelopmental outcomes and clinical risk factors will be tested using multivariable regression. Individual deviation from normal brain development will be quantified using Bayesian modelling and will be associated with neurodevelopmental outcomes.

**Ethics and dissemination:**

This study was approved by the National Health Service Health Research Authority’s ethical committee (REC reference: 22/PR/1587). Findings from this study will be published in peer-reviewed journals, presented at national and international conferences and shared with patients and their families for widespread dissemination of the results.

**STRENGTHS AND LIMITATIONS OF THIS STUDY**

- This is the first study to comprehensively investigate neurodevelopmental outcomes and associations with brain alterations in a well-powered sample of patients with early-onset chronic liver disease (CLD).
- Detailed assessment of neurodevelopmental outcomes, psychiatric comorbidities, quality of life and medical history will characterise the longer-term outcomes of patients with early-onset CLD.
- Complementary multimodal neuroimaging, including MRI, magnetic resonance spectroscopy, electroencephalography and novel eye tracking, will delineate the complex brain structure-function relationships and unravel neuronal biomarkers of dysfunction.
- Cerebral MRI will be conducted in a specific subtype of CLD, namely Biliary Atresia, to delineate the neurologic underpinnings of neurodevelopmental sequelae in this population
- Potential risk of recruitment bias may occur as those who experience neurodevelopmental difficulties may be more likely to consent and only participants with sufficient English language knowledge can be assessed.

## Introduction

 The impact of a severe physical condition is rarely restricted to one organ. Instead, organ dysfunction early in life may have a systemic impact and subsequently interfere with critical periods of brain development. This can potentially predispose to neurodevelopmental and mental health sequelae and is the focus of our study. Our exemplar of the systemic impact of organ dysfunction is early-onset chronic liver disease (CLD). Preliminary evidence suggests that children with early-onset CLD are at increased risk of non-specific neurodevelopmental or educational difficulties and poorer mental health compared with the general population.^1^,^3^ However, no study has systematically characterised the neurodevelopmental and mental health phenotype(s) associated with early-onset CLD, nor explored their possible neurobiological underpinnings.

Early-onset CLD refers to a diagnosis of ongoing liver disease diagnosed within the first 6 months of life and encompasses a spectrum of aetiologies, such as infectious, metabolic, genetic or idiopathic diseases. These lead to irreversible change in the hepatic structure and complications like cirrhosis.^4^ Biliary atresia (BA), one of the most common early-onset CLD, is a progressive liver disease characterised as a destructive, inflammatory cholangiopathy of the bile ducts with a perinatal onset. BA is a life-threatening condition requiring surgical intervention (Kasai Portoenterostomy) within the first months of life to restore bile flow out of the liver and delay further progress of the liver disease. Despite surgical intervention, half of patients with BA will subsequently require a liver transplantation by 10 years of age.^5^,^7^ Other liver conditions presenting during infancy with cholestasis and inflammation include genetic disorders such as α1-antitrypsin deficiency, progressive familial intrahepatic cholestasis (PFIC) and Alagille syndrome. While these conditions do not require surgical intervention, some patients will develop end-stage liver disease and require liver transplantation.^4^ Overall, improvements in surgical strategies—primarily the availability of liver transplantation but also advances in medical management for patients with early-onset CLD—have significantly increased survival rates in these children,^8^ leading to a shift in perspective towards their long-term well-being.

Increasing evidence suggests that individuals with early-onset CLD have a higher risk of neurodevelopmental difficulties, including lower cognitive, executive and motor functioning compared with the general population.^1^,^3^ We have also reported that there are higher than expected mental health problems in young adults^9^ and our preliminary investigations have identified a higher likelihood of autistic traits in young children with BA, with 30% of the study population receiving a clinical or research diagnosis of autism.^10^ In our local setting, there is also concern from parents and patients about possible attention-deficit/hyperactivity disorder (ADHD), autism and related difficulties. However, despite these important first steps to expose neurodevelopmental difficulties, compromised academic outcomes, poor mental health and quality of life,^111^,^14^ definitive characterisation of neurodevelopmental and mental health problems is needed: (i) to drive better clinical provision beyond liver services for this population and (ii) to identify underlying neurobiological mechanisms and inform tailored interventions.

The neurologic underpinnings of any relationship between neurodevelopmental and psychiatric difficulties and early-onset CLD remain poorly understood and are further complicated by interactions between disease chronicity and severity. The liver is essential for multiple vital processes, including hormonal regulation, metabolic functions, haematological homeostasis, detoxification and excretion.^15^ Chronic liver dysfunction may disrupt these processes, leading to toxicity and inflammation which can impact on the brain. Importantly, CLD leads to impaired clearance of toxins such as bilirubin and ammonia and, together with alterations in blood-brain barrier permeability, can lead to hepatic encephalopathy.^16 17^ Its mildest form, known as ‘minimal hepatic encephalopathy’, is characterised by subtle motor and cognitive impairments without overt clinical signs of encephalopathy (eg, disorientation or coma).^18^ Furthermore, toxins may also disrupt the brain developmental trajectory, which progresses alongside the clinical course of early-onset CLD. Importantly, studies have suggested that neurodevelopmental difficulties are already apparent in early life, prior to surgical interventions^19^,^22^ and persist post-transplant, despite the restoration of normal liver function.^23 24^ These findings indicate an early effect on the developing brain at least partially discrete from the trajectory of liver disease itself, with possibly shared aetiological factors which influence both brain and liver. However, the clinical progression of liver dysfunction with episodes of hyperammonaemia, inflammation and bilirubin toxicity, and the early life stress due to hospitalisation and medical interventions, may additionally burden the developing brain with complex consequences on brain maturation.^25^ ^26^

To untangle these influences on brain maturation, we will examine neurobiology in young people with early-onset CLD using multimodal neuroimaging, cognitive and behavioural phenotyping. There have been two prior neuroimaging studies in children with various types of CLD which have reported neurochemical and structural brain alterations, particularly in those with mild cognitive impairments, compared with healthy controls.^18 27^ However, these studies predominantly included CLD types which typically manifest in later childhood or adolescence (eg, autoimmune liver disease^28^). Consequently, it remains unclear how early-onset CLD and brain maturation is linked.

To understand this relationship, we recognise that both macrostructural and microstructural maturation occur in the perinatal and infant periods, and follow non-linear and region-specific trajectories: Macrostructurally, rapid cortical folding and white and grey matter growth occur in late pregnancy and early childhood, followed by a grey matter decline and continued white matter growth in later childhood and adolescence; microstructurally, axonal tract development begins in late pregnancy, followed by network refinement through pruning and myelination, which peaks in the neonatal period and continues into early adulthood.^29^,^32^ The etiological and progressive insults which accompany early onset CLD may then disrupt critical developmental trajectories at multiple levels, potentially interfering with both macro- and microstructural brain maturation. Moreover, the interaction between serious physical illness and other key confounders of brain development, such as family socioeconomic background^33^ or family history of neurodevelopmental conditions,^34^ remains poorly understood. To parse this complexity will demand significant investigation and resources. As crucial first steps, we will adopt multimodal neuroimaging paired with in-depth neurodevelopmental and mental health assessments to begin to delineate the complex brain structure-function-phenotype relationship and advance our understanding of the processes contributing to neurodevelopmental sequelae in early-onset CLD. Longer-term, this may improve evaluation of needs and inform the search for more tailored intervention targets for these young people.

### Aims and hypotheses

This study aims to describe the extent and spectrum of neurodevelopmental and mental health difficulties in children, adolescents and young adults with early-onset CLD. It seeks to elucidate phenotypic associations with underlying brain alterations and liver disease markers.

Aim 1: To compare neurodevelopmental and psychiatric phenotypes between patients with early-onset CLD, neurotypical controls and neurodivergent controls without CLD.

Hypothesis 1.1A: Individuals with early-onset CLD will have poorer cognitive functioning, more autistic- and ADHD-related traits, and more depression and anxiety symptoms compared with neurotypical controls.

Hypothesis 1.1B: There will be both similarities and differences in the neurodevelopmental profiles of individuals with early-onset CLD and neurodivergent controls.

Hypothesis 1.2: Mental health problems will be more prominent in those who screen positive for neurodevelopmental conditions.

Hypothesis 1.3: Within the group of individuals with early-onset CLD, neurodevelopmental outcomes will be related to liver disease indices including liver function and surgical factors. Based on our previous work in BA,^35^ we hypothesise that successful early intervention (eg, early jaundice clearance) will be associated with better general cognitive neurodevelopmental outcomes but not neurodivergent traits.

Hypothesis 1.4: Within the group of individuals with early-onset CLD, autistic/ADHD traits will be associated with parental autistic/ADHD traits and socioeconomic background. We will explore the relative contributions of family history and organ disease to neurodevelopmental outcomes.

Aim 2: (A) To compare neuroimaging markers (electroencephalography (EEG), eye-tracking and MRI) between patients with early-onset CLD, neurotypical controls and neurodivergent controls. (B) To describe individual deviation from typical brain development in patients with early-onset CLD. (C) To correlate neuroimaging markers with neurodevelopmental outcomes and clinical disease factors.

Hypothesis A.2.1: EEG. Patients with early-onset CLD will have altered event-related potentials (ERP) and functional connectivity in social vs non-social tasks and resting state EEG. Eye-tracking: Patients with early-onset CLD will have altered gaze patterns during social and attentional tasks. MRI: Patients with early-onset CLD will have alterations in brain morphology, white matter integrity, structural and functional network connectivity and brain metabolism.

Hypothesis A.2.2: There will be similarities and differences in neuroimaging markers (EEG, eye-tracking and MRI) between individuals with early-onset CLD and neurodivergent controls.

Hypothesis B.2.1: There will be individual deviation of patients with early-onset CLD from typical brain development using normative Bayesian modelling.

Hypothesis C.2.1: Neuroimaging markers, particularly those with significant group differences identified in Hypothesis A.2.1, will correlate significantly with more neurodevelopmental problems and clinical disease indices in patients with early-onset CLD.

## Methods and analysis

### Study design

**Figure 1:**
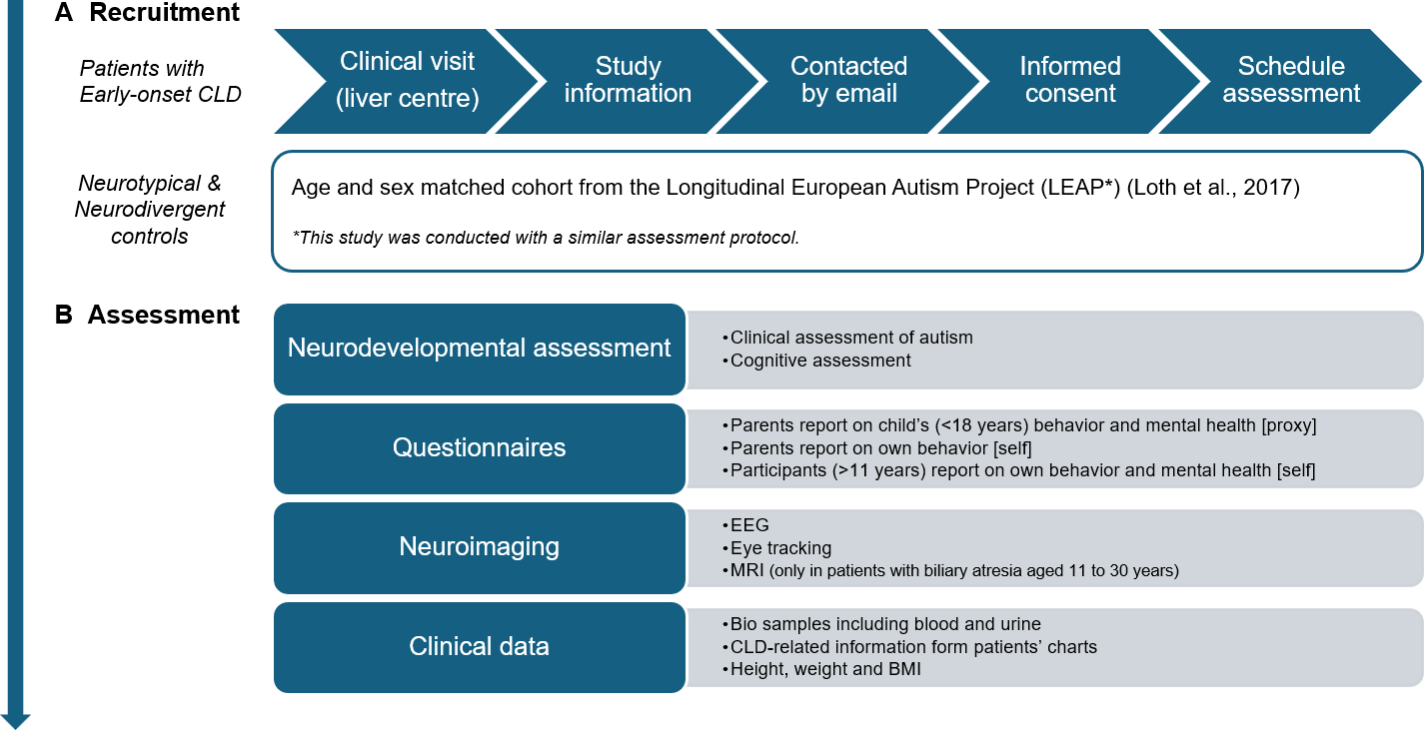
Study design. CLD, chronic liver disease.

This study assesses neurodevelopmental, psychiatric and neuroimaging data in individuals with early-onset CLD (see overview in figure 1). Data of individuals with early-onset CLD will be age and sex matched with an existing dataset of neurotypical controls and neurodivergent controls without a CLD originating from the Longitudinal European Autism Project (LEAP).^36^ Neurodivergent controls will be included in this study as a reference group, as a previous study has shown an increased risk of autistic traits in children with early-onset CLD.^10^ Neurodivergent controls have a confirmed autism diagnosis and will serve as a positive control group with neurodevelopmental difficulties but without CLD. The current study and the LEAP study use a similar assessment protocol to facilitate data pooling.

### Study population

Individuals with early-onset CLD who are treated at the paediatric and young adult liver centre at King’s College Hospital will be eligible. Early-onset CLD is defined as the presence of liver disease before 6 months of age and for a duration of more than 6 months. Early-onset CLD encompasses a wide spectrum of disorders of various aetiologies such as infectious, metabolic, genetic or of unknown aetiology diagnosed in a supra-regional paediatric liver centre. Inclusion criteria for participation in this study includes:

1. Diagnosed with CLD before 6 months of age (ie, early-onset CLD).
2. Aged between 6 and 30 years old at the time of assessment.
3. No major neurological conditions such as brain trauma associated with severe developmental delay or epilepsy.
4. No known genetic/syndromal forms of autism.
5. No significant hearing or visual impairments not corrected by glasses or hearing aids.
6. Sufficient knowledge of the English language to provide informed consent, to conduct neurodevelopmental assessments and to fill in questionnaires.

Preterm born children with early-onset CLD will be eligible for this study and gestational age at birth will be investigated as a covarying factor.

Eligible patients with early-onset CLD will be approached during one of their regular appointments at the liver centre. Study information will be provided, and if they express interest, they will be contacted via email afterwards. Written informed consent will be obtained prior to participation in accordance with the declaration of Helsinki.

### Outcome measures

On the day of assessment (see figure 1), participants and their parents will complete a set of questionnaires (30–45 min) and undergo a comprehensive neurodevelopmental evaluation, including a clinical assessment of autism (45 min) and a cognitive assessment (60 min). Subsequently, neuroimaging measures will be acquired, including EEG (45 min), eye tracking (60 min) and MRI (45 min). A lunch break will be provided between assessments according to the participant’s preference. Biological samples (blood and urine) will be collected from patients during their routine clinical visit closest to the study date, typically on the same day. CLD-related clinical data, obtained as part of standard hospital procedures, will be retrieved retrospectively from patient medical records.

#### Questionnaires

A wide battery of validated, age-appropriate questionnaires will be completed by parents and/or participants depending on their age. These will be used to gather information on social communication and repetitive behaviours in everyday life, neurodevelopmental or psychiatric comorbidities (including ADHD, depression and anxiety), quality of life, health, and life events (table 1). Age-adjusted summary scores will be calculated based on the manuals where applicable.

**Table 1:** Questionnaires stratified by respondent and age group

Participants age	30 years		11–17 years		6–10 years
Respondent	Parent	Self	Parent	Self	Parent
Social communication and repetitive behaviours					
Social Responsiveness Scale – second edition^84^	✓	✓	✓	✓	✓
Repetitive Behaviour Scale – Revised^85^	✓		✓		✓
Short Sensory Profile^86^	✓		✓		✓
Child Routine Inventory^87^	✓		✓		✓
Adult Routine Inventory^87^		✓			
Sensory Experiences Questionnaire^88 89^	✓		✓		✓
Neurodevelopmental and psychiatric comorbidities					
DSM-5 ADHD Checklist^90^	✓	✓	✓		✓
Strengths and Difficulties Questionnaire^91^	✓	✓	✓	✓	✓
Beck Anxiety Inventory^92^		✓	✓	✓	
Beck Depression Inventory^93^		✓	✓	✓	
Highly Sensitive Person Scale^94^	✓	✓	✓	✓	✓
Toronto Alexithymia Scale^95^		✓		✓	
Affective Reactivity Index^96^	✓	✓	✓	✓	✓
Cognitive Emotion Regulation Questionnaire^97^		✓		✓	
Developmental Coordination Disorder Questionnaire^98^			✓		✓
Quality of life					
Child-Health and Illness Profile^99^			✓		✓
WHO Quality of Life^100^		✓			
Columbia Impairment Scale^101^	✓	✓	✓	✓	✓
Health Information					
Demographic and medication questionnaire	✓	✓	✓		✓
Child Sleep Habits Questionnaire^102^					✓
Sleep Habits Questionnaire (adapted from^102^)		✓	✓	✓	
Edinburgh Handedness Questionnaire^103^		✓		✓	✓
Brief Diet Questionnaire		✓	✓		✓
Life events					
Brief Life Events Questionnaire^104^	✓	✓	✓		✓
Parental neurodevelopmental traits					
Social Responsiveness Scale – second edition^84^	✓		✓		✓
DSM-5 ADHD Checklist^90^	✓		✓		✓

ADHD, attention-deficit/hyperactivity disorder.

#### Neurodevelopmental assessment

##### General neurodevelopment

Cognitive functions will be assessed with the *Wechsler Abbreviated Scale of Intelligence* 2nd edition for individuals between 6 and 30 years of age.^37^ Two verbal subscales (*Vocabulary* and *Similarities*) and two non-verbal subscales (*Block Design* and *Matrix Reasoning*) will be assessed, and an IQ score is calculated based on age-adjusted norms. In addition, the subscales *Digit Span* to measure working memory, and *Symbol Search* to measure processing speed will be assessed from the *Wechsler Intelligence Scale for Children 5th edition* or the *Wechsler Adult Intelligence Scale 4th edition *depending on the participant’s age.^38 39^

##### Autistic traits

The *Autism Diagnostic Observation Schedule Second Edition* (*ADOS-2*) will be conducted to assess social and communicative behaviours associated with autism.^40^ The observation will be conducted by a trained researcher at the research site and will be recorded on video. The video will be rated by two independent examiners according to the manual. The ratings will be compared, and if discrepancies arise, a consensus discussion will be conducted.

### EEG

EEG will be recorded to international standards^41^ using a 64-channel Brain Products BrainAmp DC system equipped with ActiCap active electrodes (Brain Products GmbH, Munich, Germany). Electrode placement will adhere to the 10–20 layout, with FCz used as the recording reference. Data will be initially sampled at 5 kHz and subsequently down-sampled for analysis. Impedances will be maintained below 20 kΩ prior to data recording. Both task-based and resting-state EEG will be collected.

Indices of face processing will be measured using a face ERP task, in which participants passively view a sequence of upright and inverted faces, and upright houses.^42^ Data will be filtered and cleaned before forming individual and grand average ERPs representing brain activity time-locked to the stimuli. Key variables will be P1, N170, P2 and N2 amplitude and latency. These are neural correlates of the component stages of face processing, from early pre/attentional visual processing (P1) to face- and expertise-specialised cortex in the fusiform gyrus (N170) to higher-order cognitive components indexing processes such as recognition and categorisation (P2, N2).^42 43^

Indices of social processing will be measured using two repetitions each of a social (a woman telling nursery rhymes) and non-social (toys being operated) video for a total of 2 min.^44 45^ In addition, 2 min of eyes-closed resting state data will be acquired. After filtering, segmenting and cleaning the data, a variety of metrics will be extracted including power and connectivity in each canonical frequency band, as well as metrics related to excitation/inhibition balance such as 1 /f slope and complexity.^46^,^48^

### Eye tracking

Eye tracking data will be acquired from a Tobii TX-300 (Tobii Technology, Stockholm, Sweden) at a sampling rate of 120 Hz. Visual stimuli will be presented on an integrated 23-inch (1920×1080 pixels) screen with a 16:9 aspect ratio. Participants will be seated in front of the eye tracker and, for optimal data quality, positioned in the centre approximately 55–65 cm distance from the screen. A 5-point calibration will be performed. Data will be acquired, annotated and stored by in-house Matlab scripts via the Tobii Analytics SDK 3.0. We will present a battery of tasks:

#### Biological motion

‘Point light display’ (PLD) stimuli, representing the joints of limbs, are animated. Participants will view a paired visual preference task with two PLDs on either side of the screen with biological versus non-biological motion (rotating in place or scrambled). An attentional bias to biological motion is present from birth and conserved across species.^49 50^ Proportion of fixations, peak fixation duration and time to first fixation will be calculated per area of interest (AOI) and trial. These metrics indicate attentional preference to social stimuli, reflecting neurodevelopmental processes associated with social cognition and motion perception. A reduced preference for biological motion has been associated with higher autism-related traits.^51^

#### Passive social tasks

Indices of social attention will be measured using passive viewing of social stimuli which have been used in previous studies in neurodivergent and neurotypical individuals from infancy to adulthood.^52^,^56^ Participants will view (1) dynamic scenes of human interactions and activities (*naturalistic videos*); (2) photographs of individuals, groups and social scenarios (*static social images*); and (3) arrays of faces and non-social objects (*face popout task*). The face pop-out stimuli are equidistant from the centre of the screen, allowing for precise measurement of time to first fixation and initial preference for faces. For all tasks, comparable metrics will be extracted, including proportion of fixations, fixation duration and time to first fixation to AOIs (eg, faces, eyes, background, etc). These metrics reflect attentional allocation and sensitivity to social stimuli, providing insights into neurodevelopmental processes related to social cognition, comprehension, joint attention and engagement.

#### Gap overlap task

This task measures the speed of covert attentional shifts from a central to a peripheral stimulus.^57^ Attentional modulation depends on stimulus timing: the central stimulus disappears before (gap), remains during (overlap) or disappears simultaneously with the peripheral stimulus (baseline). Saccadic reaction times are summarised by condition, with ‘facilitation’ (gap vs baseline) and ‘disengagement’ (overlap vs baseline) scores, reflecting the benefits and costs of attentional shifts in the gap and overlap.

#### Implicit false belief

A puppet theatre scene is presented using a theory of mind paradigm, where an actor holds a false belief about the location of a ball that was moved by a puppet in their absence (scene from^58 59^). Visual patterns, including the first fixation on the correct box, will be evaluated.

#### Event memory

Five sequences of a short story followed by a matching visual scene shown for 20 s are presented. Participants are asked to memorise the visual scene and recall it afterwards. Answers are coded based on accuracy and relevance to the story. Eye tracking metrics include fixation duration on AOIs in relation to memory performance.

### Cerebral MRI

MRI will be performed on a 3 Tesla GE MR scanner with an 8-channel head coil in a subsample of patients with BA between 11 and 30 years of age. This will provide a more homogenous subsample with a similar time of disease onset, clinical course and interventional strategies (eg, Kasai surgery). It will also optimise data quality (eg, less movement artefacts in older children) and maximise budget efficiency while achieving adequate power. Suitability for MRI acquisition will be assessed using a safety-screening questionnaire, which will be filled out by the parents or participants >16 years. For the MRI scan, hearing protection will be provided including earplugs and headsets. Participants can watch a movie during the scan except during resting-state fMRI. Total scanning time will take approximately 45 min. Participants can communicate with the radiographer via a microphone and headphones and may press an emergency button at any time to stop the scan. To investigate cortical morphometry, brain volumes and the presence of macroscopic brain abnormalities, high-resolution three-dimensional T1-weighted images will be acquired using a magnetisation-prepared rapid gradient echo sequence with 1 mm slice thickness and prospective motion correction sequence. Cortical reconstruction and volumetric segmentation will be conducted with Freesurfer (https://surfer.nmr.mgh.harvard.edu/). Total and regional brain volumes, gyrification index, surface area and cortical thickness will be quantified. In addition, vertex-wise morphometry will be conducted to investigate regional differences in cortical morphology. Subcortical volumes will also be estimated with the same package. To investigate macrostructural abnormalities like white matter injuries, a T2-weighted Fluid-attenuated inversion recovery (FLAIR) will be performed. T2-weighted and T1-weighted structural images will be reviewed by a trained researcher for macroscopic brain abnormalities and incidental findings (eg, global or focal atrophy, white matter lesions, microhaemorrhages, stroke, cysts and oedema). Clinically relevant brain abnormalities are communicated to the participant’s clinical team and/or general practitioner (GP) who would then support any further referrals needed.

To investigate microstructural properties and structural network connectivity, spin echo diffusion MRI will be performed with 2 cm slice thickness, a total of 60 diffusion-weighted gradient directions, six interleaved non-diffusion weighted images with b=0 s/mm² and diffusion-weighted images with b=1500 s/mm². To enable distortion correction, a second acquisition will be acquired with the same acquisition settings except the phase-encoding direction will be reversed, and only six gradient directions will be acquired. A standard processing pipeline will be used to conduct artefact correction (denoising, Gibbs-ringing, eddy current, motion and slice to volume corrections), calculate whole-brain voxel-wise scalar maps of fractional anisotropy, mean diffusivity and radial diffusivity and finally register into standard space.^60^,^62^ For network analyses, constrained spherical deconvolution will be used to estimate weighted, undirected connectome matrices based on nodes defined by an anatomical atlas and edges defined by sum of weighted streamlines.^60^

To investigate functional network properties and connectivity, resting-state fMRI will be conducted with 3 mm slice thickness. Patients will be asked to look at a fixation cross during acquisition. A 3D field map will be acquired to enable distortion correction of the rs-fMRI scan. Multi-echo scans will be recombined using echo-time weighted averaging. A standard processing pipeline will be used to conduct artefact correction, standard space registration, spatial smoothing, independent component analysis to identify spatially independent resting-state networks and dual regression to obtain subject-specific spatial maps and mean time series. For network analyses, weighted connectome matrices will be estimated based on nodes defined by an anatomical atlas and edges defined by correlation coefficients.

To investigate the profile of brain metabolites, magnetic resonance spectroscopy (240 transients) will be acquired with a voxel (24×40×26 mm) placed in the thalamus using a Hadamard Encoding and Reconstruction of MEGA-edited spectroscopy (HERMES) sequence.^63^ HERMES allows for the quantification of neurometabolite levels in the living brain. The thalamus has been chosen as a region of interest due to its crucial role in subcortical-cortical integration and sensory processing^64^—which are frequently impaired in neurodevelopmental conditions.^65^ Further, HERMES in the thalamus is particularly useful to investigate GABA and Glx which are key markers to investigate excitatory inhibitory (E/I) imbalance. E/I imbalance has previously been linked to neurodevelopmental conditions.^66^ In addition, other brain metabolites that have been linked to minimal hepatic encephalopathy will also be investigated, including myoinositol (glia and osmoregulation marker), choline (cell membrane components) and N-Acetylaspartate (a neuronal marker) or glutathione.^18 67 68^ Additional metabolites associated with hormonal profiles may be investigated exploratively. The Osprey software^69^ will be used for analysis and data will be reported in line with minimum reporting standard consensus.^70^

### Clinical data

Clinical data, which are obtained as part of standard hospital procedures, will be collected retrospectively from patients’ medical charts including information from ultrasound scans. In addition, bio samples from blood and urine will be collected from patients at the time of assessment. Clinical data of interest include markers for liver function (ie, bilirubin, blood count, gamma-glutamyl transferase, albumin, aspartate and alanine aminotransferase), renal function (ie, urea, creatinine, sodium, potassium), vitamin levels (A, D, K), cytokine profiles, immunosuppression and autoantibodies (for those with liver transplantation), steroid hormone profile and growth. These markers will be compiled at the time of diagnosis; 1-, 3- and 6 months post-diagnosis; at liver transplantation; 1-, 3- and 6 months post-transplantation (if applicable); and at the time of assessment. In addition, birth demographics and surgery-related factors (if applicable) will be collected. The predictive value of clinical variables for brain development and neurodevelopmental outcomes will be investigated.

### Statistical analyses

#### Sample size and power calculations

Based on the feasibility of recruiting patients from our clinic, we aim to recruit 100 patients with BA and an additional 50 patients with other types of early-onset CLD, such as α1-antitrypsin deficiency, PFIC and Alagille syndrome. This approach will enable us to explore similarities and differences between subtypes of early-onset CLD and investigate risk factors associated with diagnosis and clinical course and their relationship with neurodevelopmental outcomes. Considering that each year approximately 40 children are diagnosed with early-onset CLD at King’s College Hospital, this proposed sample size is feasible.

Of the 100 patients with BA, those patients who are aged between 11 and 30 years and meet the MR safety requirements will additionally undergo cerebral MRI. For MRI analyses, a moderate effect size d=0.5, α=0.05, ß=0.9 and total n=36 is required for group comparison and normative modelling, which assumes similar effect sizes as previous studies in other autism risk cohorts (eg, gestational diabetes). This is likely under-estimated considering that the majority of children with CLD demonstrate neurodevelopmental difficulties.^1^ To adjust for multiple testing, we will recruit n=72 patients with BA (50% transplant, 50% native liver). This corresponds to an α=0.01.

Neurotypical controls and neurodivergent controls have been assessed previously as part of the LEAP study.^36^ Neurodevelopmental and neuroimaging data of 222 neurotypical controls and 307 neurodivergent controls (individuals with autism without CLD) is available. The sample size of neurotypical controls is similar to previous studies conducting normative modelling.^71^,^73^ In total, we aim to include 150 patients with early-onset CLD (100 patients with BA, 50 patients with other types of early-onset CLD), 222 neurotypical controls and 307 neurodivergent controls.

#### Data analysis plan

Sample descriptives stratified by group (patients, neurotypical controls, neurodivergent controls) will be reported. For aim 1, neurodevelopmental and psychiatric outcomes will be compared with normative means (when available), as well as to the means of neurotypical control and neurodivergent control groups, utilising parametric and non-parametric tests as appropriate. Proportions of individuals with clinically relevant scores (>SD from normative mean or above clinical threshold, as appropriate) will be reported and compared between groups using χ^2^ tests. Post hoc, we will also compare patients with BA to other forms of early-onset CLD to explore disease-specific phenotypes. Associations with clinical risk factors will be explored using multivariable regression analysis.

For aim 2, neuroimaging metrics will be investigated on a group level and on an individual level. For group-level analyses, neuroimaging markers will be compared between patients with early-onset CLD, neurotypical controls and neurodivergent controls using multivariable regression models. Furthermore, associations between neuroimaging markers, neurodevelopmental outcomes and clinical risk factors will be tested using multivariable regression models. All analyses will be controlled for age and sex. For individual-level analyses, normative brain developmental curves will be built from neuroimaging data of neurotypical individuals using non-parametric Bayesian regression analysis—similar to the growth curves used in clinical practice.^71^ Estimates and predictive confidence of neuroimaging metrics of patients with early-onset CLD will be calculated based on the normative curves to model individual patient deviation from norm. Deviation from the norm will be quantified by age- and sex-adjusted z-scores which can then be correlated with neurodevelopmental outcomes and clinical risk factors. Post hoc, we will also explore multimodal metrics, including associations between diffusion and functional MRI, to gain a deeper understanding of the structural and functional relationships in brain network alterations, as well as associations between resting-state EEG and functional MRI to optimise temporal and spatial resolution.

### Patient and public involvement statement

This study arises in response to the concerns of patients and their parents attending our clinical services, namely that difficulties, for example in school and employment, were not addressed. We have also worked closely with the Childhood Liver Disease Charity who have similar concerns. The study design and the procedures aligns with a large-scale study of brain development in autistic and non-autistic individuals (AIMS-2-TRIALS) and autistic individuals (A-reps) collaborated to advise on protocol and ethics considerations. The results from this study will be disseminated to participants and their families by means of newsletters, patient organisation platforms and public informative meetings. Specifically, patient and public involvement and engagement (PPIE) initiatives linked to this study will include educating current and future patients and their families on study results and relevant topics including neurodevelopmental difficulties, mental health, education, clinically relevant findings from neuroimaging, etc, and the setup of focus groups including patients, parents/carers, and healthcare professionals to discuss future clinical and research priorities.

## Ethics and dissemination

The protocol was approved by the ethical committee of the NHS Health Research Authority, UK (REC reference: 22/PR/1587). Written informed consent will be obtained from participants aged 16 years or older and from parents or legal guardians of participants younger than 16 years of age. Study participants will not receive any monetary compensation for participating in the study but will be reimbursed for lunch and travel. Data handling, record keeping and archiving will be done according to ethics guidelines and the data protection and information security guidelines. To ensure a widespread dissemination of study findings, the results of this study will be presented at national and international conferences; published in international peer-reviewed journals; and presented to parent organisations, healthcare stakeholders, patients and their families.

## Discussion

This study aims to comprehensively describe the neurodevelopmental and psychiatric phenotype of children, adolescents and young adults with early-onset CLD and to elucidate phenotypic associations with underlying brain alterations and liver disease markers.

This research is driven by the concerns of patients and their parents/carers related to difficulties in school, employment and daily functioning. These stakeholders will continue to be integral to the research, engaged with planning, delivery, interpretation and dissemination. Although our study is carried out for research, we will be utilising clinical tools such as the ADOS and other standardised measures. Following the wishes of our patients and their families, the results will be available should a patient warrant further clinical referral to neurodevelopmental and/or mental health services. This aspect of our work will also be followed up to understand the rates of ‘conversion’ of research findings to formal diagnostic confirmation. This in turn will inform the development of tailored clinical pathways. Furthermore, the observational study design may provide an opportunity for a comprehensive follow-up assessment of neuroimaging and outcome measures to study developmental and psychiatric trajectories through adolescence and young adulthood.

Considering the current overload of mental health services and long waitlists,^74^ as well as the complex medical needs of patients with CLD, specialised clinical pathways providing a holistic care model including medical, neurodevelopmental and mental health care are likely to be needed—similar to existing care models in preterm born children.^75 76^ In addition, patients may need non-medical interventions, including support at school with reasonable adjustments and psychological support coordinated with local services. Psychoeducation for all stakeholders, including patients, their families, liver specialists, GPs and teachers, will be essential to facilitating timely referrals for neurodevelopmental assessments and ensuring effective treatment implementation. This study represents an initial step towards informing these activities and will, we hope, encourage the development and evaluation of clinical pathways for diagnostics and interventions. Longer-term, this should include assessing the safety and effectiveness of common neurodevelopmental and mental health treatments in this population.

Taking a broader perspective, neurodevelopmental and mental health difficulties are not restricted to patients with early-onset CLD, but a consensus is emerging that they are common across different early-onset chronic conditions, such as congenital heart disease, cystic fibrosis, paediatric kidney disease and paediatric organ transplant.^77^,^83^ In addition, comparing early-onset liver disease with cases presenting later in childhood—whether in acute settings or with later-onset chronic conditions—may provide insight into how the timing of disease onset influences sensitive brain developmental processes and the extent to which disease-specific pathologies contribute to long-term outcomes. The design of this study may serve as a template for investigating various chronic conditions, which even if rare, are cumulatively common. This would facilitate transdiagnostic comparisons to enhance our understanding of shared and disease-specific mechanisms that contribute to altered brain development trajectories and neurodevelopmental difficulties in these conditions and support access to interventions perhaps previously unavailable.
