# Supplementary material for: Investigating brain maturation, neurodevelopmental and psychiatric outcomes in individuals with early-onset liver disease: protocol of a single-centre observational study
Source: BMJ Open. 2025 Nov 26;15(11):e103290. doi: 10.1136/bmjopen-2025-103290 (PMC12658490; doi:10.1136/bmjopen-2025-103290)
Supplement: Supplementary file 1 [file bmjopen-2025-103290.reviewer_comments.pdf]

PEER REVIEW HISTORY

BMJ Open publishes all reviews undertaken for accepted manuscripts. Reviewers are asked to complete a checklist review form and are provided with free text boxes to elaborate on their assessment. These free text comments are reproduced below.

ARTICLE DETAILS

Title (Provisional)

Investigating Brain Maturation, Neurodevelopmental and Psychiatric Outcomes in Individuals with Early-onset Liver Disease: Protocol of a Single-centre Observational Study

Authors

Ehrler, Melanie; Megan, Earl; Jemma, Day; O'Muircheartaigh, Jonathan; Mason, Luke; Nicolaas, Puts; Flavio, Dell'Acqua; Blackmore, Charlotte E.; McAlonan, Grainne; Marianne, Samyn

---

VERSION 1 - REVIEW

**Reviewer**	**1**
**Name**	**Christensen, Vibeke**
**Affiliation**	**University of Copenhagen, Dept of Pediatrics**
**Date**	**28-Apr-2025**
**COI**	**None**

I wish to congratulate the authors with this work. The protocol is well written and has a high academic level. The research question is relevant. The study is well designed and the chosen cohorte and number of participants acceptable.

The inclusion criteria are well defined and the chosen methods to evaluate brain maturation are of high standards.

**Reviewer**	**2**
**Name**	**A McLin, Valérie**
**Affiliation**	**Geneva University Hospitals**
**Date**	**18-Jul-2025**
**COI**	**Consulting for Ipsen Pharmaceuticals**

Consulting for Mirum Pharmaceuticals

This study is potentially of great significance, as it addresses a subject that has been little explored or documented in pediatrics. To the best of our knowledge it is the first time neurodiverse controls will be used. Additionally, it will shed light on the neurometabolic and morphological changes related to neurological, neurocognitive, and psychiatric outcomes of patients having developed CLD in infancy and childhood, which may also be relevant in other chronic conditions.

Minor issues :

1. If premature babies are included in the study, how will their neurological and psychological development be assessed given that they usually differ from the general population -- this may influcence results?

Major questions-- related to methods

2. Why will MRS be performed in the thalamus? Give rationale

3. mIns—not just a glial marker, but also a marker of osmoregulation (ref Cudalbu McLin PMID: 37356555)

4. Will brain edema be assessed and how?

4. Clinical data: will peek bilirubin, or peek NH3 be extracted from charts? and used to stratify according to disease severity. Since this is a prospective study, consider obtaining plasma aminoacids: The plasma Fischer ratio (molar ratio BCAA/

AAA) decreases in patients with chronic liver disease. In the

study of adults by Campollo et al,17 mean values for various

types of subjects were as follows: normal individuals (3.9),

stable cirrhotics (2.9), unstable cirrhotics (1.7), and patients

with HE (0.8). An imbalance in plasma levels of AAA phenylalanine,

tyrosine, and tryptophan and BCAAs and their

BCAA/AAA ratio has been suggested to play a causal role in

HE by enhanced brain AAA uptake and subsequently disturbed

neurotransmission (from Foerster AJNR 30 Sep 2009)

5. Cerebral MRI is observational- does not offer mechanistic insight (therefore would caution against use of the term 'mechanistic?

6. Hypothesis 1.1B- based on their vast experience in a high volume center, do the authors have an idea where the similarities and differences might lie?

7. Hypothesis 1.4 is not a hypothesis

8. Aim 2C- what is meant by brain alterations- metabolic? Structural?

9. Hypothesis A.2.2: hypothesis about which markers similar or different?

10. Hypothesis C2.1_ not clear

11. Neurodiverse controls: autism in itself is a very broad group. While clearly the LEAP study cohort is interesting and a huge opportunity, I find that it is a surprising control group- what is the prevalence of ASD in the CLD cohort at KCL?

12. There is a need for a Gantt chart or figure to illustrate study- a bit muddled – will the patients undergo longitudinal f/u? Not clear how data will be compared—are the patients in LEAP undergoing the same imaging or neurodevelopmental assessment protocol?

13. It seems that patients with congenital portosystemic shunts would be excellent controls, as the problem with assessing the neurocognitive /neuropsychological diversity of patients with CLD is that is is unclear what is due to the liver disease and what is secondary to portosystemic shunting. Knowing that KCL has a sizeable cohort of patients with congenital portosystemic shunts, these would seem a very appropriate control group to adjust for intrinsic liver disease --

All in all, this is a very interesting and ambitious project. The study itself could benefit from some methodological improvement and the present manuscript from increased granularity. Thank you for the opportunity to read this very intersting project, and to contribute in a small way through the review process.

VERSION 1 - AUTHOR RESPONSE

Reviewer 1

I wish to congratulate the authors with this work. The protocol is well written and has a high academic level. The research question is relevant. The study is well designed and the chosen cohort and number of participants acceptable. The inclusion criteria are well defined and the chosen methods to evaluate brain maturation are of high standards.

Response: We thank the reviewer for their encouraging feedback on our study protocol.

Reviewer 2

This study is potentially of great significance, as it addresses a subject that has been little explored or documented in pediatrics. To the best of our knowledge it is the first time neurodiverse controls will be used. Additionally, it will shed light on the neurometabolic and morphological changes related to neurological, neurocognitive, and psychiatric outcomes of patients having developed CLD in infancy and childhood, which may also be relevant in other chronic conditions.

Response: We thank the reviewer for the positive evaluation of our manuscript and for the important critical comments. We have addressed all the comments below and believe that this has greatly helped to improve the manuscript.

1. If premature babies are included in the study, how will their neurological and psychological development be assessed given that they usually differ from the general population -- this may influcence results?

Response: We agree with the reviewer that prematurity may influence neurologic and psychological development additionally. Prematurity is not an exclusion criteria for this study. If preterm born individuals are included in our study sample, we will explore gestational age at birth as a covarying factor in our statistical models.

Please see changes in the manuscript section 3.2 (page 8): "<u>Preterm born children with early-onset CLD will be eligible for this study and gestational age at birth will be investigated as a covarying factor</u>."

2. Why will MRS be performed in the thalamus? Give rationale

Response: We thank the reviewer for their comment and appreciate the opportunity to expand on the rational of MRS analyses in the thalamus. First, we are limited to a single voxel as MRS is time-consuming, and we are interested in a broader spectrum of neuroimaging measures. We chose the thalamus because of the following reasons:

1. <u>Methodological consistency and comparability</u>: Acquiring thalamic MRS maintains methodological consistency with the study protocol of our healthy and neurotypical controls: The AIMS-2-TRIALS longitudinal European Autism Project (LEAP). This allows a direct comparison of metabolite profiles of patients and controls.
2. <u>Central role in sensory processing</u>: We chose the thalamus as it is a primary sensory relay station, making it directly relevant to your sensory processing outcomes. Thalamic nuclei filter, integrate, and modulate sensory information before cortical processing, with GABAergic interneurons playing crucial roles in sensory gating mechanisms. This is of particular interest as sensory processing difficulties are common across neurodevelopmental conditions. Beyond sensory processing, thalamic processes as a hub connecting cortical and subcortical regions makes it particularly informative for understanding circuit-level dysfunction and it contributes significantly to higher-order behaviours.
3. <u>Disrupted brain development</u>: The thalamus undergoes substantial development throughout childhood and adolescence, with protracted maturation of both glutamatergic neurons (excitatory) and GABAergic interneurons (inhibitory). An excitatory/inhibitory imbalance has previously been discussed as a key pathology in individuals with autism (Rubenstein et al., 2003; Hollestein et al., 2023). We are interested if thalamic dysfunction and related excitatory/inhibitory imbalance may disrupt thalamocortical circuits essential for normal development.

We have now added a rational to our manuscript.

Please see changes in the manuscript section 3.3.5 last paragraph (page 14): "HERMES allows for the quantification of neurometabolite levels in the living brain. <u>The thalamus has been chosen as region of interest due to its crucial role in subcortical-cortical integration and sensory processing [84] – which are frequently impaired in neurodevelopmental conditions [85]. Further, HERMES in the thalamus</u> is particularly useful to investigate GABA and Glx which are key markers to investigate excitatory inhibitory (E/I) imbalance. E/I imbalance has previously been linked to neurodevelopmental conditions [86]."

[84] Mease RA, Krieger P, Groh A. Cortical control of adaptation and sensory relay mode in the thalamus. Proceedings of the National Academy of Sciences. 2014;111:6798–803. doi: 10.1073/pnas.1318665111
[85] Scheerer NE, Pourtousi A, Yang C, et al. Transdiagnostic Patterns of Sensory Processing in Autism and ADHD. J Autism Dev Disord. 2024;54:280–92. doi: 10.1007/s10803-022-05798-3

Additional references:

Rubenstein JLR, Merzenich MM. Model of autism: increased ratio of excitation/inhibition in key neural systems. Genes Brain Behav. 2003;2:255–67.

Hollestein, Viola, et al. "Excitatory/inhibitory imbalance in autism: the role of glutamate and GABA gene-sets in symptoms and cortical brain structure." Translational Psychiatry 13.1 (2023): 18.

3. mIns—not just a glial marker, but also a marker of osmoregulation (ref Cudalbu McLin PMID: 37356555)

Response: Thank you for making us aware of this link. We have added this information and the respective paper to our manuscript.

Please see changes in the manuscript section 3.3.5 last paragraph (page 14): "In addition, other brain metabolites that have been linked to minimal hepatic encephalopathy (mHE) will also be investigated, including myoinositol (glia and osmoregulation marker), choline (cell membrane components), and N-Acetylasparate (NAA, a neuronal marker) or glutathione [27,84,87]."

[87] Cudalbu C, Xin L, Marechal B, *et al.* High field brain proton magnetic resonance spectroscopy and volumetry in children with chronic, compensated liver disease – A pilot study. *Anal Biochem*. 2023;675. doi: 10.1016/j.ab.2023.115212

4. Will brain edema be assessed and how?

Response: Thank you for this important question. The likelihood of our patients having overt hepatic encephalopathy and edema is slim, as we are not including patients with acute liver failure in our study and we focus on patients diagnosed before the age of six months of life with chronic liver disease. We acknowledge however that the patients who have not undergone liver transplantation and who have evidence of portal hypertension and/or more advanced liver disease are at risk of developing or having minimal hepatic encephalopathy (MHE) which is known to be associated with brain edema. We will use a T2-weighted FLAIR sequence, which can be helpful in detecting edema and we will also assess water diffusion using diffusion tensor imaging (DTI) which has been shown to correlated with MHE in children with chronic liver disease (Srivastava et al., 2017). In addition, all MRI scans will be investigated by a trained researcher or radiologist to rule out any major abnormalities.

Reference: Srivastava, Anshu, et al. "Minimal hepatic encephalopathy in children with chronic liver disease: prevalence, pathogenesis and magnetic resonance-based diagnosis." Journal of hepatology 66.3 (2017): 528-536.

Please see changes in the manuscript section 3.3.5 second paragraph (pages 13-14) : "T2-weighted and T1-weighted structural images will be reviewed by a trained researcher for macroscopic brain abnormalities and incidental findings (e.g., global or focal atrophy, white matter lesions, microhaemorrhages, stroke, cysts, and edema)."

4. Clinical data: will peek bilirubin, or peek NH3 be extracted from charts? and used to stratify according to disease severity. Since this is a prospective study, consider obtaining plasma aminoacids: The plasma Fischer ratio (molar ratio BCAA/AAA) decreases in patients with chronic liver disease. In the
study of adults by Campollo et al, 17 mean values for various types of subjects were as follows: normal individuals (3.9), stable cirrhotics (2.9), unstable cirrhotics (1.7), and patients with HE (0.8). An imbalance in plasma levels of AAA phenylalanine, tyrosine, and tryptophan and BCAAs and their

BCAA/AAA ratio has been suggested to play a causal role in HE by enhanced brain AAA uptake and subsequently disturbed neurotransmission (from Foerster AJNR 30 Sep 2009)

Response: Thank you for highlighting the importance of specific blood markers related to liver disease and brain development. In clinic, we do not routinely monitor plasma amino acids in our patients with chronic liver disease mainly related to cost constraints. Serum ammonia levels are occasionally checked in selective patients with significant portal hypertension and those listed for liver transplantation. However, we are aware of its limitations in the context of timing of taking and processing samples in the laboratory. Therefore, we are not able to include amino acids and ammonia levels in our study.

We have shown in a previous study in infants with biliary atresia that serum bilirubin levels 1-month post Kasai surgery (i.e., an indicator of jaundice clearance) is associated with long-term neurodevelopmental outcomes (Earl et al., 2025). We will retrospectively collect bilirubin levels, other liver function markers, clotting profile and full blood count from patient charts at multiple time points including at diagnosis, at the time of surgery (Kasai or LTx), 1-, 3-, and 6-months post-surgery, and at the study assessment. This will allow us to examine how the timing and duration of liver dysfunction relate to neurodevelopmental outcomes and brain development. We have now added a list of blood markers of interest to our protocol.

Reference: Earl, M., Samyn, M., Blackmore, C., Brace, M., Day, J., Javed, A., ... & McAlonan, G. M. (2025). General and Autism-Related Neurodevelopmental Difficulties in Biliary Atresia. The Journal of Pediatrics, 114673.

Please see changes in the manuscript section 3.3.6 (page 15): "Clinical data of interest include markers for liver function (i.e., bilirubin, blood count, gamma-glutamyl transferase, albumin, aspartate and alanine aminotransferase), renal function (i.e., urea, creatin, sodium, potassium), vitamin levels (A, D, K), cytokine profiles, immunosuppression and autoantibodies (for those with liver transplantation), steroid hormone profile and growth."

5. Cerebral MRI is observational- does not offer mechanistic insight (therefore would caution against use of the term 'mechanistic?

Response: Thank you for this comment. We agree that the term "mechanistic" might be misleading and have revised this accordingly.

Please see changes in the manuscript:

Section Article summary (page 3): "Cerebral MRI will be conducted in a specific subtype of CLD, namely Biliary Atresia, to delineate the neurologic underpinnings of neurodevelopmental sequelae in this population"

Section 1 fourth paragraph (page 5): "The neurologic underpinnings of any relationship between neurodevelopmental and psychiatric difficulties and early-onset CLD remain poorly understood and are further complicated by interactions between disease chronicity and severity."

6. Hypothesis 1.1B- based on their vast experience in a high volume center, do the authors have an idea where the similarities and differences might lie?

Response: Thank you for this comment. As this is the first study comparing neurodevelopmental and psychiatric outcomes in patients with early-onset CLD and neurodivergent controls, no prior evidence is available. However, in a previous study of 35 infants with biliary atresia, 30% scored above the clinical threshold for autism (Earl et al., 2025). In addition, another study from our group found that individuals scoring above the autism threshold also exhibited higher anxiety levels (manuscript in preparation), a link that has also been reported in general autism research (Van Steensel et al., 2011). Further, we

know that in general, autistic and ADHD traits are highly comorbid (Rong et al., 2021). However, no study has investigated the prevalence of ADHD and comorbidities with autism traits in early-onset CLD.

Since not all patients with early-onset CLD display neurodevelopmental traits, we anticipate that their neurodevelopmental profiles may diverge from those of neurodivergent controls to some extent. We believe that our study will provide important evidence in this regard, but, given the lack of existing data, we refrain from making specific assumptions.

References:

Earl, M., Samyn, M., Blackmore, C., Brace, M., Day, J., Javed, A., ... & McAlonan, G. M. (2025). General and Autism-Related Neurodevelopmental Difficulties in Biliary Atresia. The Journal of Pediatrics, 114673.

Van Steensel, F. J., Bögels, S. M., & Perrin, S. (2011). Anxiety disorders in children and adolescents with autistic spectrum disorders: A meta-analysis. Clinical child and family psychology review, 14(3), 302-317.

Rong, Y., Yang, C. J., Jin, Y., & Wang, Y. (2021). Prevalence of attention-deficit/hyperactivity disorder in individuals with autism spectrum disorder: A meta-analysis. Research in Autism Spectrum Disorders, 83, 101759.

7. Hypothesis 1.4 is not a hypothesis

Response: Thank you for pointing this out. We have changed the phrasing accordingly.

Please see changes in the manuscript section 2 (page 8): "Within the group of individuals with early-onset CLD, autistic/ADHD traits will be associated with parental autistic/ADHD traits and socioeconomic background. We will explore the relative contributions of family history and organ disease to neurodevelopmental outcomes."

8. Aim 2C- what is meant by brain alterations- metabolic? Structural?

Response: Thank you for this comment. We changed this term to "neuroimaging markers" to keep it more general and discuss the specific markers in the hypotheses.

Please see changes in the manuscript section 2 (page 7): "To correlate neuroimaging markers with neurodevelopmental outcomes and clinical disease factors."

9. Hypothesis A.2.2: hypothesis about which markers similar or different?

Response: Considering that this is the first study investigating a broad range of neuroimaging modalities (EEG, eye-tracking, and MRI) in patients with early-onset CLD and comparing these to neurodivergent individuals, there is no previous evidence we can rely on to make any assumptions about specific directions of effects.

10. Hypothesis C2.1_ not clear

Response: We have revised this hypothesis for better clarity.

Please see changes in the manuscript section section 2 (page 7): "Neuroimaging markers, particularly those with significant group differences identified in Hypothesis A2.1, will correlate significantly with more neurodevelopmental problems and clinical disease indices in patients with early-onset CLD**."**

11. Neurodiverse controls: autism in itself is a very broad group. While clearly the LEAP study cohort is interesting and a huge opportunity, I find that it is a surprising control group- what is the prevalence of ASD in the CLD cohort at KCL?

Response: Thank you for this important comment. We have preliminary data from a study involving 35 infants with biliary atresia, where 30% scored above the clinical threshold on the Autism Diagnostic Observation Schedule (ADOS), a gold standard for diagnosing autism (Earl et al., 2025). Additionally, we assessed an autism screening questionnaire in 160 children and adolescents with different types of liver disease (early and later onset, acute and chronic), and 9% scored above the threshold for autism with those being diagnosed within the first year of life being at highest risk (manuscript in preparation). These findings suggest an increased likelihood for autistic traits in patients with early-onset CLD. However, well-powered studies using more comprehensive neurodevelopmental and psychiatric assessments are still lacking, and the current study protocol aims to address this gap. We have now added a rational for including neurodivergent controls to the methods section.

Please see changes in the manuscript section 3.1 (page 8): "Neurodivergent controls will be included in this study as a reference group, as a previous study has shown an increased risk of autistic traits in children with early-onset CLD [10]."

[10] Earl, M., Samyn, M., Blackmore, C., Brace, M., Day, J., Javed, A., ... & McAlonan, G. M. (2025). General and Autism-Related Neurodevelopmental Difficulties in Biliary Atresia. The Journal of Pediatrics, 114673.

12. There is a need for a Gantt chart or figure to illustrate study- a bit muddled – will the patients undergo longitudinal f/u? Not clear how data will be compared—are the patients in LEAP undergoing the same imaging or neurodevelopmental assessment protocol?

Response: Thank you for this comment. We have provided an overview of the study design in Figure 1 in the original version of this manuscript. The current protocol is only cross-sectional. However, this study cohort may provide a valuable opportunity for follow-up. A few participants might be on the wait list for a liver transplantation and post-transplant follow up might be of interest. Also, trajectories of brain development through adolescence and young adulthood might be of interest. However, this is beyond the scope of the current study protocol. We have added an outlook to our discussion section.

The current study uses the same assessment protocol as LEAP. This has been indicated in Figure 1 and is described in section 3.1.

Please see changes in the manuscript

Section 3.1 (page 8): "The current study and the LEAP study use a similar assessment protocol to facilitate data pooling."

Section 5 second paragraph (page 18): "Furthermore, the observational study design may provide an opportunity for a comprehensive follow-up assessment of neuroimaging and outcome measures to study developmental and psychiatric trajectories through adolescence and young adulthood."

13. It seems that patients with congenital portosystemic shunts would be excellent controls, as the problem with assessing the neurocognitive /neuropsychological diversity of patients with CLD is that is unclear what is due to the liver disease and what is secondary to portosystemic shunting. Knowing that KCL has a sizeable cohort of patients with congenital portosystemic shunts, these would seem a very appropriate control group to adjust for intrinsic liver disease

Response: Thank you for this suggestion. We agree that patients with congenital portosystemic shunts could serve as an additional comparison group. Currently, we have received funding and ethics approval to study patients with CLD diagnosed within the first six months of life, as a core focus of our work is the long-term impact of early organ disease on brain development. In the future, we are keen to explore other childhood-onset diagnoses including patients with congenital portosystemic shunts

as well as those with extrahepatic portal vein occlusion and acute liver failure - which are typically associated with a variable degree of hepatic encephalopathy. In the discussion, we have already considered expanding the project to other early-onset chronic conditions, and we have now added a statement about comparing these to later-onset and acute conditions to better understand the impact of disease timing and the extent to which other disease-specific pathologies – such as portosystemic shunting – contribute to long-term outcomes.

Please see changes in the manuscript section 5 last paragraph (page 18): "In addition, comparing early-onset liver disease with cases presenting later in childhood – whether in acute settings or with later-onset chronic conditions – may provide insight into how the timing of disease onset influences sensitive brain developmental processes and the extent to which disease-specific pathologies contribute to long-term outcomes. The design of this study may serve as a template for investigating various chronic conditions, which even if rare, are cumulatively common."

All in all, this is a very interesting and ambitious project. The study itself could benefit from some methodological improvement and the present manuscript from increased granularity. Thank you for the opportunity to read this very interesting project, and to contribute in a small way through the review process.

Response: We thank the reviewer for their valuable comments and the positive encouragement regarding our study.

General comment

1) Please note that we have changed the reference for a preprint to the now published article.
   Old: Earl M, Samyn M, Blackmore C, *et al.* General and autism-related neurodevelopmental difficulties in Biliary Atresia. *SSRN Preprint*. 2024;1–19. doi: 10.2139/ssrn.5004494

   New: Earl M, Samyn M, Blackmore C, et al. General and Autism-Related Neurodevelopmental Difficulties in Biliary Atresia. J Pediatr. 2025;285:114673. doi: 10.1016/j.jpeds.2025.114673

2) Please note that we have changed the term "neurodiverse controls" to neurodivergent controls" throughout the manuscript, as neurodivergent specifically relates to individuals with neurodevelopmental traits.

VERSION 2 - REVIEW

**Reviewer**	**2**
**Name**	**A McLin, Valérie**
**Affiliation**	**Geneva University Hospitals**
**Date**	**11-Oct-2025**
**COI**	

All in all the authors have addressed most of this reviewer's concerns. It is a very ambitious study, with many confounders which will need to be taken into account- some more challenging than others. I did not find an 'alternative approaches' section, but maybe beyond the scope of a protocol?

major points:

-It would be worthwhile to make a link with hepatic encephalopathy in the background and rationale section: in essence this is what they are exploring in kids. The clinical expression is different owing to neurodevelopment. This is how chronic liver disease differs from other systemic diseases - liver disease is by definition associated with circulating neurotoxins.

- How will the 'subset' of patients with BA be chosen to undergo MRI MRS? How long do the acquisitions last?

- are the patients compensated for their participation in the study?

- how will clinically relevant findings identified on MRI be shared with the subjects/patients? (p.13)

minor points:

-on p.8: 3. Study population vi) they state that sufficient English knowledge is required for consent. What about to answer the numerous questionnaires? This may be a limitation of the study given the international population in London where the study will be conducted.

- how long will patients/subjects be in the MRI and can they get out should they need?

- I would caution against the use of 'associated' in the methods f.ex. 'associated with neurodevelopmental outcomes' rather 'multivariate analysis will be used to assess for any association with neurodevelopmental outcomes'.

- Figure 1: although this helps clarify, the timing is unclear

- clinical data: specify that it is retrospective, and per KCL protocol

---

VERSION 2 - AUTHOR RESPONSE

Reviewer 2

All in all the authors have addressed most of this reviewer's concerns. It is a very ambitious study, with many confounders which will need to be taken into account- some more challenging than others. I did not find an 'alternative approaches' section, but maybe beyond the scope of a protocol?

Response: Thank you for evaluating our revised manuscript. According to the BMJ Open – Study protocol guidelines, the description of 'alternative approaches' is not required but a description of the planned study design should be provided.

Guideline in [Authors | BMJ Open]: *"provide a full description of the study design, including the following. How the sample will be selected; interventions to be measured; the sample size calculation*

(drawing on previous literature) with an estimate of how many participants will be needed for the primary outcome to be statistically, clinically and/or politically significant; what outcomes will be measured, when and how; a data analysis plan."

-It would be worthwhile to make a link with hepatic encephalopathy in the background and rationale section: in essence this is what they are exploring in kids. The clinical expression is different owing to neurodevelopment. This is how chronic liver disease differs from other systemic diseases - liver disease is by definition associated with circulating neurotoxins.

Response: Thank you for this important comment. We have included a statement about the role of mild hepatic encephalopathy and potential interactions with brain development.

Please see changes in the manuscript section 1 (page 5): "Importantly, CLD leads to impaired clearance of toxins such as bilirubin and ammonia and, together with alterations in blood-brain barrier permeability, can lead to hepatic encephalopathy [16,17]. Its mildest form known as 'minimal hepatic encephalopathy' is characterized by subtle motor and cognitive impairments without overt clinical signs of encephalopathy (e.g., disorientation or coma) [18]. Furthermore, toxins may also disrupt the brain developmental trajectory, which progresses alongside the clinical course of early-onset CLD."

[18] Srivastava A, Chaturvedi S, Kumar Gupta R, et al. Minimal hepatic encephalopathy in children with chronic liver disease: Prevalence, pathogenesis and magnetic resonance-based diagnosis. J Hepatol. 2017;66:528–36. doi: 10.1016/j.jhep.2016.10.026

- How will the 'subset' of patients with BA be chosen to undergo MRI MRS? How long do the acquisitions last?

Response: Thank you for this comment. The subset undergoing MRI and MRS includes patients with BA who are between 11 and 30 years of age and meet the safety requirements for MRI (e.g., no ferromagnetic implants). We have clarified this in the manuscript.

Please see changes in the manuscript section 3.4.1 (page 16): "Of the 100 patients with BA, those patients, who are aged between 11 and 30 years and meet the MR safety requirements, will additionally undergo cerebral MRI."

- are the patients compensated for their participation in the study?

Response: Study participants do not receive monetary compensation but will be reimbursed for travel and lunch.

Please see changes in the manuscript section 4 (page 18): "The protocol was approved by the ethical committee of the NHS Health Research Authority, UK (REC reference: 22/PR/1587). Written informed consent will be obtained from participants aged 16 years or older and from parents or legal guardians of participants younger than 16 years of age. Study participants will not receive any monetary compensation for participating in the study but will be reimbursed for lunch and travel."

- how will clinically relevant findings identified on MRI be shared with the subjects/patients? (p.13)

Response: We thank the reviewer for this important question. As outlined in Section 3.5 ("Patient and Public Involvement Statement"), sharing clinically relevant study findings with patients and their families is addressed through our PPIE initiatives. In addition, if a clinically relevant finding is identified in an individual participant, the principal investigator of this study will directly communicate these findings to their clinical care team and/or GP. This process follows the guidelines of our institute.

Please see changes in the manuscript

Section 3.5 (page 17): "The results from this study will be disseminated to participants and their families by means of newsletters, patient organisation platforms and public informative meetings.

Specifically, patient and public involvement and engagement (PPIE) initiatives linked to this study will include educating current and future patients and their families on study results and relevant topics including neurodevelopmental difficulties, mental health, education, clinically relevant findings from neuroimaging etc, and the setup of focus groups including patients, parents/carers, and healthcare professionals to discuss future clinical and research priorities."

Section 3.3.5 (page 14): "T2-weighted and T1-weighted structural images will be reviewed by a trained researcher for macroscopic brain abnormalities and incidental findings (e.g., global or focal atrophy, white matter lesions, microhaemorrhages, stroke, cysts, and edema). Clinically relevant brain abnormalities are communicated to the participant's clinical team and/or general practitioner (GP) who would then support any further referrals needed."

on p.8: 3. Study population vi) they state that sufficient English knowledge is required for consent. What about to answer the numerous questionnaires? This may be a limitation of the study given the international population in London where the study will be conducted.

Response: Thank you for this comment. We agree that language barriers can be a limitation of psychological questionnaires and neurodevelopmental assessments. We have clarified this in the manuscript and added this limitation.

Please see changes in the manuscript

Article summary (page 3): "Potential risk of recruitment bias may occur as those who experience neurodevelopmental difficulties may be more likely to consent and only participants with sufficient English language knowledge can be assessed."

Section 3.2 Inclusion criteria (page 8): "Sufficient knowledge of the English language to provide informed consent, to conduct neurodevelopmental assessments, and fill in questionnaires."

- how long will patients/subjects be in the MRI and can they get out should they need?

Response: The MRI/MRS protocol takes approximately 45 minutes. We follow the standard safety protocol. Participants can press an emergency button at any time if they wish to stop the scan and exit the scanner. The radiographers communicate with participants in between every sequence (i.e., approximately every 2 to 8 minutes)

Please see changes in the manuscript section 3.3.5 (page 13): "For the MRI scan, hearing protection will be provided including earplugs and headsets. Participants can watch a movie during the scan except during resting-state fMRI. Total scanning time will take approximately 45 minutes. Participants can communicate with the radiographer via a microphone and headphones and may press an emergency button at any time to stop the scan."

- I would caution against the use of 'associated' in the methods f.ex. 'associated with neurodevelopmental outcomes' rather 'multivariate analysis will be used to assess for any association with neurodevelopmental outcomes'.

Response: Thank you for this comment. As suggested, we have rephrased the term "associated with".

Please see changes in the manuscript:

Abstract (page 2): "Associations with clinical risk factors will be explored using multivariable regression. Neuroimaging markers will be compared between groups and associations with neurodevelopmental outcomes and clinical risk factors will be tested using multivariable regression."

Section 3.4.2 (page 17): "For aim 1, neurodevelopmental and psychiatric outcomes will be compared to normative means (when available), as well as to the means of neurotypical control and neurodivergent control groups, utilizing parametric and non-parametric tests as appropriate. Proportions of individuals with clinically relevant scores (>standard deviation from normative mean or above clinical threshold, as appropriate) will be reported and compared between groups using χ^2 tests.

Post-hoc, we will also compare patients with BA to other forms of early-onset CLD to explore disease specific phenotypes. Associations with clinical risk factors will be explored using multivariable regression analysis.

For aim 2, neuroimaging metrics will be investigated on a group-level and on an individual-level. For group-level analyses, neuroimaging markers will be compared between patients with early-onset CLD, neurotypical controls and neurodivergent controls using multivariable regression models. Furthermore, associations between neuroimaging markers, neurodevelopmental outcomes and clinical risk factors will be tested using multivariable regression models. All analyses will be controlled for age and sex."

- Figure 1: although this helps clarify, the timing is unclear

Response: Thank you for pointing this out. We have added a brief description of the assessment day, to clarify the assessment procedure and timing.

Please see changes in the manuscript section 3.3 (page 9): "On the day of assessment (see Figure 1), participants and their parents will complete a set of questionnaires (30–45 minutes) and undergo a comprehensive neurodevelopmental evaluation, including a clinical assessment of autism (45 minutes) and a cognitive assessment (60 minutes). Subsequently, neuroimaging measures will be acquired, including EEG (45 minutes), eye tracking (60 minutes), and MRI (45 minutes). A lunch break will be provided between assessments according to the participant's preference. Biological samples (blood and urine) will be collected from patients during their routine clinical visit closest to the study date, typically on the same day. CLD-related clinical data, obtained as part of standard hospital procedures, will be retrieved retrospectively from patient medical records."

FIGURE 1: Study design

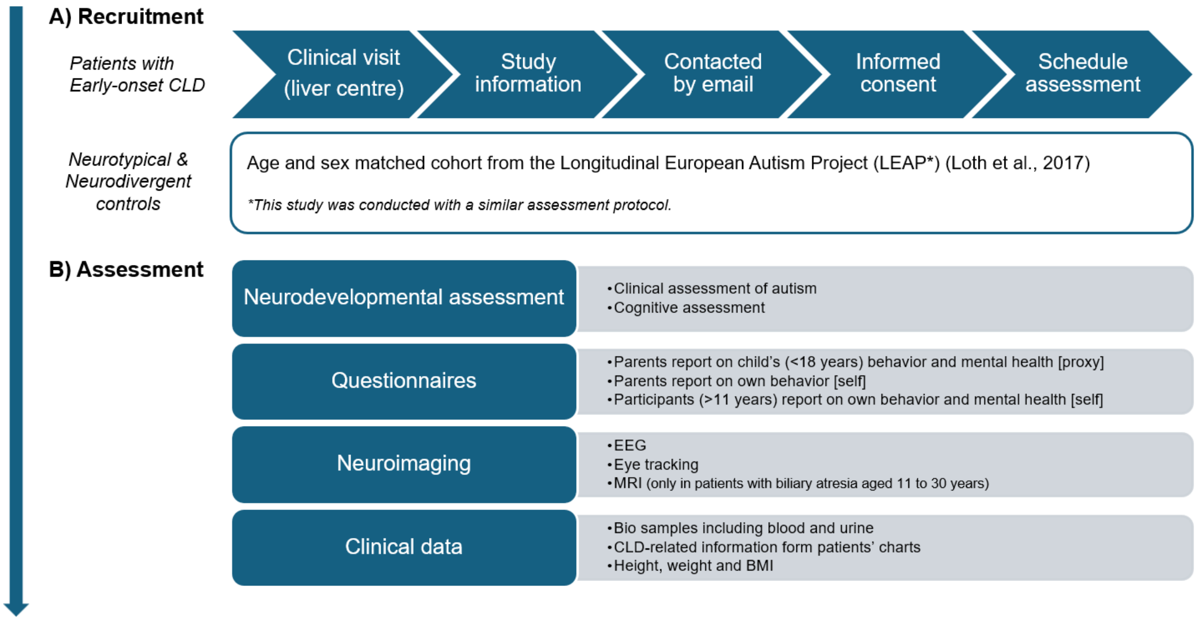

- clinical data: specify that it is retrospective, and per KCL protocol

Response: Thank you for this comment. We have clarified this in our manuscript as suggested.

Please see changes in the manuscript

Section 3.3 (page 9): "CLD-related clinical data, obtained as part of standard hospital procedures, will be retrieved retrospectively from patient medical records."

Section 3.3.6 (page 15): "Clinical data, which are obtained as part of standard hospital procedures, will be collected retrospectively from patients' medical charts including information from ultrasound scans. In addition, bio samples from blood and urine will be collected from patients at the time of assessment."
